# Effect of *Ganoderma lucidum* to Produce Functional Chocolate: Physicochemical, Textural and Sensory Properties

**DOI:** 10.1002/fsn3.70676

**Published:** 2025-09-16

**Authors:** Yasamin Zahra Bazyar, Mohammad Rabbani, Mohammad Hossein Azizi

**Affiliations:** ^1^ Department of Food Science and Technology, North Tehran Branch Islamic Azad University Tehran Iran; ^2^ Department of Food Science and Technology, Faculty of Agriculture Tarbiat Modares University Tehran Iran

**Keywords:** anticancer activity, chocolate, functional foods, *Ganoderma lucidum*

## Abstract

Functional chocolate was developed by incorporating various concentrations (0%, 5%, 10%, 15%, and 20%) of *Ganoderma lucidum* (GL). Proximate chemical analysis, total phenolic content (TPC) and total flavonoid content (TFC), as well as antioxidant and antitumor activity of chocolate samples, were measured at day 1 and 4th month of storage. To evaluate the oxidative stability during storage, peroxide value (PV), thiobarbituric acid reactive substances (TBARS), and acid value were measured. Color indexes and texture hardness were measured as quality criteria. Nutritional analysis revealed that fiber, moisture, fat, and protein contents ranged from 0.45% to 5.32%, 0.32% to 0.34%, 34.09% to 36.13%, and 0.43% to 13.4%, respectively. At 20% GL concentration, TPC and TFC reached 68.23 and 201.1 mg/g, respectively. The antioxidant activity of GL‐enriched chocolate was measured at 63.69 μg/mL, showing improved oxidative resistance. Additionally, MTT assay results indicated that increasing GL concentration enhanced antitumor activity. Color analysis showed a concentration‐dependent decrease in lightness (L*) and yellowness (b*), with an increase in redness (a*). Sensory evaluation demonstrated a decline in texture, sweetness, and color preferences at higher GL levels, although 5% GL retained good acceptability. These findings suggest that 5% GL is a promising additive for the development of functional chocolate, combining health‐promoting properties with acceptable sensory qualities.

## Graphical abstract

This study explores the incorporation of *Ganoderma lucidum* (GL) in chocolate formulation and its impact on physicochemical characteristics, oxidative stability, nutritional composition, and bioactive properties. Our findings suggest that GL, particularly at 5% concentration, has strong potential as a functional ingredient, enhancing both the nutritional and health‐promoting attributes of chocolate.

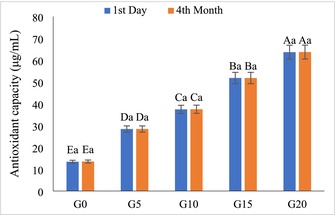

## 1 Introduction

The International Food Information Council defines functional foods (FFs) as foods that provide health benefits beyond basic nutrition. FFs are categorized into six major groups: functional proteins, functional carbohydrates, functional lipids, prebiotics and probiotics, isoprenoids, and phenolic compounds, as well as minerals and micronutrients (Balcázar‐Zumaeta et al. 2023; Sugumar and Guha 2020). The enrichment of FFs is achieved by incorporating nutrients or bioactive compounds, which are typically derived from plant, animal, or microbial sources (Bakshi et al. 2020; Fu et al. 2022). These foods are highly nutritious and offer various health benefits, including the prevention of nutritional deficiencies, enhancement of overall health, and protection against diseases (Tiwary and Hussain 2021). Moreover, they may reduce the risk of chronic diseases and contribute to both physical and mental well‐being (Baker et al. 2022).

Mushrooms are increasingly recognized as valuable ingredients in the development of FFs due to their rich content of bioactive compounds, including vitamins, minerals, and antioxidants, as well as their long‐standing role in both traditional diets and natural medicine (Budzyńska et al. 2022). They are excellent sources of minerals, proteins, and vitamins while being nutritious, healthy, and low in calories. The presence of B vitamins in mushrooms contributes to the maintenance of red blood cell health and the proper functioning of the nervous system (Mohamed et al. 2020).

Among these, *Ganoderma lucidum*, which has been extensively utilized in the medicinal and FF industries (Chen et al. 2022; Lu et al. 2020), is known for its triterpenoids and polysaccharides, which are among the most pharmacologically active compounds associated with *GL* (Bulam et al. 2019). Previous studies have reported its diverse bioactivities, including antihistamine (Ang et al. 2021), antihypertensive (El Sheikha 2022), antiangiogenic (Jana and Acharya 2022), antitumor and immunomodulatory (Cadar et al. 2023; Xia et al. 2021), antioxidant (Ahmad et al. 2024), anti‐inflammatory (Xu et al. 2021), antimicrobial (Erbiai et al. 2023), antidiabetic (Shao et al. 2022), and antifungal properties (Yang et al. 2022). These health benefits are largely attributed to the presence of triterpenoids, polysaccharides, nucleotides, sterols, steroids, fatty acids, and specific proteins and peptides (El Sheikha 2022; Martínez‐Montemayor et al. 2019). In recent years, GL has been successfully incorporated into various FF products, such as herbal teas, yogurt, biscuits, cereal bars, and chocolate, due to its health‐promoting potential and consumer appeal (Matijašević and Sknepnek 2024; Swallah et al. 2023).

Chocolate is a broad term referring to homogeneous products derived from a combination of cocoa derivatives, milk, additives, sugars, and/or sweeteners. The cocoa solids content varies depending on the type of chocolate, with a minimum of 25% in milk chocolate and 35% in dark chocolate. Additionally, ingredients such as starch and animal fats may be incorporated to create diverse chocolate products (Cozentino et al. 2022). The beneficial effects of antioxidants and flavanols, such as catechin, in the chocolate on human health have been well documented. Catechin helps neutralize free radicals generated by oxidative processes. Moreover, it contributes to reducing low‐density lipoprotein levels, subsequently lowering blood pressure and inhibiting platelet aggregation (Sorrenti et al. 2020). The quality of chocolate is influenced by both the quantity and type of ingredients used, as well as the manufacturing processes involved (Faccinetto‐Beltrán et al. 2021). Chocolate is widely consumed by people of all ages across the world. Its ability to evoke sensory pleasure and induce positive emotions plays a key role in its widespread popularity (Konar et al. 2022).

With the increasing prevalence of cardiovascular diseases and obesity, consumers are becoming more conscious of the ingredients used in food production. As a result, there is a growing demand for FF that offers additional health benefits beyond basic nutrition (Konar et al. 2022). The nutritional trend of the food industry in recent years has created new challenges in the field of designing and formulating new food products with medicinal, beneficial, low‐fat, low‐calorie, probiotic, prebiotic, and synbiotic properties. In other words, nowadays consumers prefer foods; in addition to being safe for them, they also have nutritional benefits. The chocolate was produced by adding different concentrations of GL. A novel approach by formulating functional chocolate enriched with GL, a medicinal mushroom known for its potent bioactive properties, will be introduced. To the best of our knowledge, no prior research has explored the development of functional chocolate incorporating GL, making this research a pioneering effort in the field. This innovative FF exhibits remarkable health‐promoting properties, including antitumor effects, which could revolutionize the functional confectionery industry. Beyond product formulation, this research provides critical insights into the feasibility and stability of *GL*‐fortified chocolate during storage. The findings can serve as a valuable reference for future applications in the food industry, paving the way for the development of functional confectionery products with enhanced health benefits.

## 2 Materials and Methods

### 2.1 Materials

GL (*Ganoderma lucidum*) was purchased from a traditional market in Tehran, Iran. Sugar and vanilla (Golha, Tehran, Iran), cacao butter (KI‐kepong, Malaysia), cacao powder (Altinmarka, Turkey), lecithin and maltodextrin (Arshihdashimi, Tehran, Iran), polyglycerol polyriconalate (PGPR) (Palsgaard, Zierikzee, Netherlands) were purchased. All chemicals were analytical grade and purchased from Merck, Germany.

### 2.2 Methods

#### 2.2.1 Preparation of GL Chocolate

GL (0.0%–20.0%), sugar (15.0%), cocoa powder (15%), cocoa butter (30.48%), maltodextrin (36.02%), lecithin (0.40%), vanilla (0.10%) and polyglycerol polyricinoleate (PGPR) (0.10%) were used in the production of GL chocolate samples. The chocolate preparation process followed the method described by Cheragheshahi et al. (2025) with slight modification. The ingredient proportions were kept constant to produce 2 kg of GL chocolate for each formulation. Initially, sugar, cocoa powder, and GL were blended using a low speed mixer and passed through laboratory sieves (20 μm). Subsequently, cocoa butter and lecithin, which had been pre‐melted in a water bath (60°C for 20 min), were added to the dry mixture and mixed at medium speed for 5 min. The resulting chocolate paste underwent refining and conching using a pilot‐scale ball mill (2 h, 45°C, 60 rpm). The melted chocolate was then cooled to 35°C and poured into pre‐warmed polycarbonate molds (35°C). After molding, the chocolate samples were cooled in a cooling chamber (10°C for 30 min), removed from the molds, wrapped in aluminum foil, and stored at 15°C until further analysis (Cheragheshahi et al. 2025).

#### 2.2.2 Proximate Chemical Analysis

The moisture content was determined using the Karl Fischer method (AOAC approach). Fat was measured by the Soxhlet apparatus with n‐Hexane solvent using the AOAC method. Protein was measured by the Kjeldahl technique.

#### 2.2.3 Determination of Fiber

This test was performed according to AOAC Method No. 10‐32. First, the sample was degreased using petroleum ether. Then, 3 g of the fat‐free sample was placed into the crucible of the Ankom device (Italy), and 200 mL of H_2_SO_4_ (0.255 N) was added. The mixture was boiled for 30 min, after which the acid was removed using vacuum filtration. Next, 200 mL of NaOH solution (0.133 N) was added to the crucible and boiled for another 30 min. The resulting mixture was then evacuated under vacuum. The remaining material in the crucible was washed multiple times with water before being placed at 100°C until a constant weight was achieved. After weighing the crucible, the sample was transferred to an oven at 550°C for incineration to ash. Once cooled, the crucible was weighed again, and the fiber content was determined (AOAC 2012).

#### 2.2.4 Determination of Total Phenolic Content

Total phenolic content (TPC) was determined by the Folin Ciocalteu reagent method using gallic acid as a standard. For this purpose, 10 mg of the sample extract was added to 3 mL of distilled water, 0.25 mL of Folin Ciocalteu, and 2 mL of Na_2_CO_3_ solution (7.5%) and placed in a hot water bath with a temperature of 37°C for 30 min. Then, the sample absorption was recorded by spectrophotometer (CM‐5 Konica Minolta, Germany) at 765 nm. Results were reported in terms of gallic acid equivalent (mg/g) as an index of phenolic compounds (Indiarto et al. 2024).

#### 2.2.5 Determination of Total Flavonoid Content

In this experiment, AlCl_3_ solution and quercetin were used as a reagent and standard flavonoid compound, respectively. For this purpose, 2.5 mL of quercetin solution with different concentrations (2, 5, 10, 20, and 40 mg/mL) was mixed with 2.5 mL of ethanol solution of AlCl_3_ with a concentration of 20 μg/mL and incubated at room temperature. After 40 min, the absorbance of each sample was recorded at 415 nm. This experiment was repeated three times for each quercetin concentration, and then the quercetin calibration curve was plotted based on the obtained absorption information. The same method was used for the samples, with the difference that the extract of the samples was used instead of quercetin. The total flavonoid content (TFC) value was determined based on quercetin in mg/g using a standard curve (Indiarto et al. 2024). The TPC of the samples was determined using Equation (1).

(1) $$\;\mathrm{TFC}=c\frac{V}{m}$$

where *c* is the concentration of quercetin obtained from the calibration curve, respectively. *V* and *m* show the volume of the extract and mass of the extract, respectively.

#### 2.2.6 Determination of Antioxidant Activity

Antioxidant activity was performed by the inhibition of free radicals using the 2,2‐diphenyl‐1‐picrylhydrazyl (DPPH) method. 0.1 mL of the desired phenolic extract was added to 3.9 mL of DPPH methanolic solution (0.0025% in methanol) in a cuvette. The reaction solution was incubated in the dark at room temperature for 60 min, and its absorbance was recorded by a spectrophotometer at 515 nm. The control sample was prepared by adding 0.1 mL of methanol to 3.9 mL of DPPH methanol solution, and its absorption was recorded at the desired wavelength. Quercetin and methanol were considered positive and negative control samples, respectively (Jaćimović et al. 2022). The calculation related to the inhibition of free radical DPPH is described using Equation (2).

(2) $$\text{Inhibition}\;\left(\%\right)=\left(\frac{A_{0}-A_{1}}{A_{0}}\right)\times 100$$

where *A*_0_ and *A*_1_ represent the absorbance of the control sample and the absorbance of the desired sample, respectively.

#### 2.2.7 MTT Assay

Glioblastoma cell line U 87 (prepared from cell bank) was cultured in a growth medium of DMEM‐F12 with 10% FBS, 100 units of penicillin–streptomycin antibiotics in an incubator at 37°C and 5% CO_2_. After three cell subcultures, the appropriate number of cells was separated for testing. The cells were trypsinized and collected from flasks, and the total number of cells was counted using a Neubauer slide. The cells were then divided into two groups, including those treated with *GL* and untreated as a control. The cells were treated with different concentrations of dark chocolate and soup, and the remaining few wells were considered as control and untreated. The plates were incubated for 24 and 48 h in a CO_2_ incubator (5%) at 37°C. Different concentrations of dark chocolate and prepared soup from 12 to 65 μg were used as serial dilution. Then 20 μL of MTT solution was added to each well. After incubation, the growth medium in the wells was drained, and 100 μL of DMSO solvent was added to each well at a wavelength of 570 nm. After several min of incubation at room temperature, their absorption was recorded at 570 nm (Mello et al. 2022).

#### 2.2.8 Determination of Peroxide Value

Lipids were extracted from chocolate samples (50 g) using a mixture of water, ethanol, and chloroform in a ratio of 25:100:100 (v/v). The PV was determined using the modified method of Kumari et al. (2021). Briefly, 1 g of fat was dissolved in 25 mL of a solvent mixture containing chloroform and citric acid (2:3). To this solution, 1 mL of saturated potassium iodide was added, and the mixture was kept in the dark for 10 min. Following this, 30 mL of distilled water and 1 mL of starch solution (1%) were added to the resulting solution, which was then titrated with 0.01 mol/L sodium thiosulfate until the solution became colorless. After that, 5 g of the sample was weighed and transferred to a 250 mL Erlenmeyer flask. Then, 30 mL of a mixture of acetic acid and chloroform (2:1) was added. Next, 0.5 mL of saturated potassium iodide was added, and the mixture was kept in the dark for 1 min. Afterward, 30 mL of distilled water and a few drops of starch solution were added. Finally, the solution was titrated with 0.01 N sodium thiosulfate until the blue color disappeared. The same procedure was also applied to the control sample (Kumari et al. 2021). The PV value was calculated using Equation (3).

(3) $$\;\mathrm{PV}\;\left(\mathrm{meq}/\mathrm{kg}\right)=\frac{S\times N}{W}\times 1000$$

where *S*, *N*, and *W* denote the volume of titration (mL), the normality of sodium thiosulfate, and sample weight (Kg), respectively.

#### 2.2.9 Determination of Thiobarbituric Acid Reactive Substances (TBARs)

The TBARS content was measured using the colorimetric method, following the correction method of Kumari et al. (2021). Approximately 10 g of the sample was weighed and homogenized with 1 mL of butylated hydroxytoluene (1 mg/mL) and 35 mL of trichloroacetic acid (5%). The resulting homogeneous solution was transferred to a flask, after which 100 mL of distilled water was added and the mixture was distilled. After collecting 50 mL of the distillate, the solution was filtered through Whatman No. 1 filter paper. Then, 5 mL of the filtered solution was mixed with 5 mL of TBA solution (0.02 M) and heated in a water bath at 100°C for 60 min. Following cooling, the absorbance was measured at 532 nm using water as the control (Kumari et al. 2021). The TBARS value was calculated using Equation (4).

(4) $$\;\text{TBARS}\;\left(\frac{\mathrm{eq}\;\mathrm{mg}}{\mathrm{Kg}}\right)=\frac{\left(\mathrm{AC}\times V\right)}{W}$$

Here AC and *W* represent the amount determined from the calibration curve and the sample weight. Also, *V* is the volume (mL) of the total extract prepared.

#### 2.2.10 Determination of Acid Value (AV)

First, 5 g of the chocolate was homogenized with twice the distilled water (g/L) and the pH of the samples was determined by pH meter at 25°C. 30 mL of neutral ethanol and 2 mL of phenolphthalein reagent were added. Then, it was titrated with NaOH (0.1 N) until a pale pink color appeared (Kumari et al. 2021).

#### 2.2.11 Determination of Hardness

Two hours before the experiment, chocolate samples were placed in a refrigerated incubator at a temperature of 20°C. Then, the hardness of the samples (25 × 40 × 5 mm) was tested by a texture analyzer machine (Santam MRT‐5, UK) equipped with a flat‐tipped mandrel with a diameter of 1.6 mm and a speed of 90 mm per min. The maximum force at a depth of 4 mm was reported as a hardness index (De Pelsmaeker et al. 2020).

#### 2.2.12 Determination of Color Index

Hunterlab device (DANATEL, CSC9203A) was used to measure the colors of the samples. First, the device was calibrated with the existing standards. Then, the samples were placed on a glass plate in a special place of the device. Afterward, the color indexes, including redness (a*), yellowness (b*), and lightness (L*) were read (Saedi et al. 2024).

#### 2.2.13 Sensory Evaluation

Sensory characteristics of chocolate samples, including sweetness, texture, melting point in the mouth, color, and general acceptance using a five‐level hedonic test (1: very bad, 2: bad, 3: moderate, 4: good, 5: very good) were evaluated by 15 panelists in the age range of 23–43 years (8 male and 7 female). Each sample was randomly assigned a three‐digit code. Samples were randomly given to the evaluators. The sensory evaluation test was performed at 0, 15, 30, 45, and 60 days after production. The difference was that this test was performed at 0 and 4 months after production (Santana et al. n.d.).

### 2.3 Statistical Analysis

The experiments were carried out using a completely randomized design, with treatments evaluated in triplicate. Analysis of variance was performed to assess the significance of the data using IBM SPSS software (V. 20.). Pairwise comparisons of samples were performed using the t‐test. Mean comparisons were conducted using Duncan's multiple range test at a 95% confidence level.

## 3 Results and Discussion

### 3.1 Proximate Chemical Analysis

The results of the effect of incorporating GL into the chocolate formulation are presented in Table 1. The moisture content remained relatively stable across all samples during the 4‐month storage period, with no statistically significant differences observed (*p* > 0.05). This suggests that the addition of GL did not affect the chocolate's moisture retention or loss, indicating good stability in terms of water content.

**TABLE 1:** The effect of *Ganoderma lucidum* on moisture, fat, protein, and fiber content of functional chocolate during storage.

Parameters (%)	Storage time	G0	G5	G10	G15	G20
Moisture	1th day	0.3 ± 20.00^Aa^	0.32 ± 0.05^Aa^	0.32 ± 0.02^Aa^	0.32 ± 0.01^Aa^	0.32 ± 0.01^Aa^
	4th month	0.33 ± 0.01^Aa^	0.34 ± 0.02^Aa^	0.31 ± 0.02^Aa^	0.31 ± 0.02^Aa^	0.34 ± 0.02^Aa^
Fat	1th day	34.0 ± 90.02^Aa^	34.15 ± 0.01^Aa^	36.13 ± 0.01^Aa^	35.84 ± 0.01^Aa^	34.13 ± 0.01^Aa^
	4th month	34.5 ± 0.01^Aa^	34.43 ± 0.02^Aa^	34.23 ± 0.01^Aa^	35.88 ± 0.02^Aa^	34.54 ± 0.02^Aa^
Protein	1th day	0.43 ± 0.06^Ae^	3.20 ± 0.01^Ad^	6.60 ± 0.01^Ac^	9.10 ± 0.01^Ab^	13.0 ± 0.01^Aa^
	4th month	0.44 ± 0.02^Ae^	3.23 ± 0.02^Ad^	6.62 ± 0.02^Ac^	9.21 ± 0.04^Ab^	13.4 ± 0.02^Aa^
Fiber	1th day	0.45 ± 0.06^Ae^	1.20 ± 0.01^Ad^	2.50 ± 0.01^Ac^	3.80 ± 0.02^Ab^	5.10 ± 0.03^Aa^
	4th month	0.46 ± 0.01^Ae^	1.34 ± 0.03^Ad^	2.43 ± 0.02^Ac^	3.87 ± 0.01^Ab^	5.32 ± 0.02^Aa^

*Note:* Different lowercase letters (Aa, Ab, Ac, Ad, Ae) indicate statistically significant differences (*p* < 0.05) between chocolates at same day. Different uppercase letters indicate statistically significant differences (*p* < 0.05) between days of storage at chocolate. G0, G5, G10, G15, and G20 are chocolate formulations containing 0%, 5%, 10%, 15%, and 20% *G. lucidum* , respectively.

Fat content showed minor variations among the samples on the first day, with the G10 sample exhibiting a slightly higher fat percentage compared to others. Over the storage period, fat content remained relatively unchanged across formulations, with no clear trend of increase or decrease. This indicates that the inclusion of GL does not negatively impact fat composition or stability in the chocolate. The study conducted by Verma and Singh (2017) revealed that incorporating oyster mushroom powder increased the protein, fat, fiber, and ash content in food products such as potato pudding. These findings align well with the current study, where the addition of GL similarly enhanced the nutritional value of the chocolate (Verma and Singh 2017).

A direct correlation was observed between the proportion of GL and the protein content of chocolate. The control sample (G0) contained a negligible amount of protein (0.43%), whereas the sample with 20% GL (G20) exhibited a significantly higher protein content (13%). This increase highlights the potential of GL as a protein‐rich ingredient that enhances the nutritional profile of chocolate. Moreover, protein content remained stable throughout storage, suggesting its structural integrity over time because mushroom is a good source of high‐quality protein (Farzana et al. 2017).

Similar to protein, fiber content increased proportionally with the addition of GL. The control sample contained only 0.45% fiber, whereas the G20 sample exhibited a substantial increase to 5.1%. This rise is attributed to the fiber‐rich nature of GL. Additionally, fiber content remained consistent throughout storage, demonstrating its stability within the chocolate matrix. The significant rise in fiber content with the addition of GL is in agreement with earlier findings reporting the high dietary fiber content of Ganoderma species. For instance, Fraile‐Fabero et al. (2021) documented a fiber content of 69.35%, whereas Nile and Park (2014) reported 35%. These high values support the increased fiber levels observed in GL‐enriched chocolates (Fraile‐Fabero et al. 2021; Nile and Park 2014).

The incorporation of GL into chocolate significantly enhanced its nutritional composition, particularly in terms of protein and fiber content. These findings suggest that GL can serve as a valuable functional ingredient for formulating nutritionally superior chocolates. Furthermore, moisture and fat content remained largely unaffected by its inclusion, ensuring product stability over time. This study highlights the feasibility of developing GL‐enriched chocolate with improved health benefits while maintaining desirable compositional stability. Rathore et al. (2019) investigated the technological, nutritional, functional, and sensorial attributes of cookies fortified with *Calocybe indica* mushroom. Their findings revealed that the addition of mushroom powder led to an increase in the fiber, protein, and fat content of the fortified cookies (Rathore et al. 2019) which is in line with the results of the present study.

### 3.2 Total Phenolic and Flavonoid Content

**FIGURE 1:**
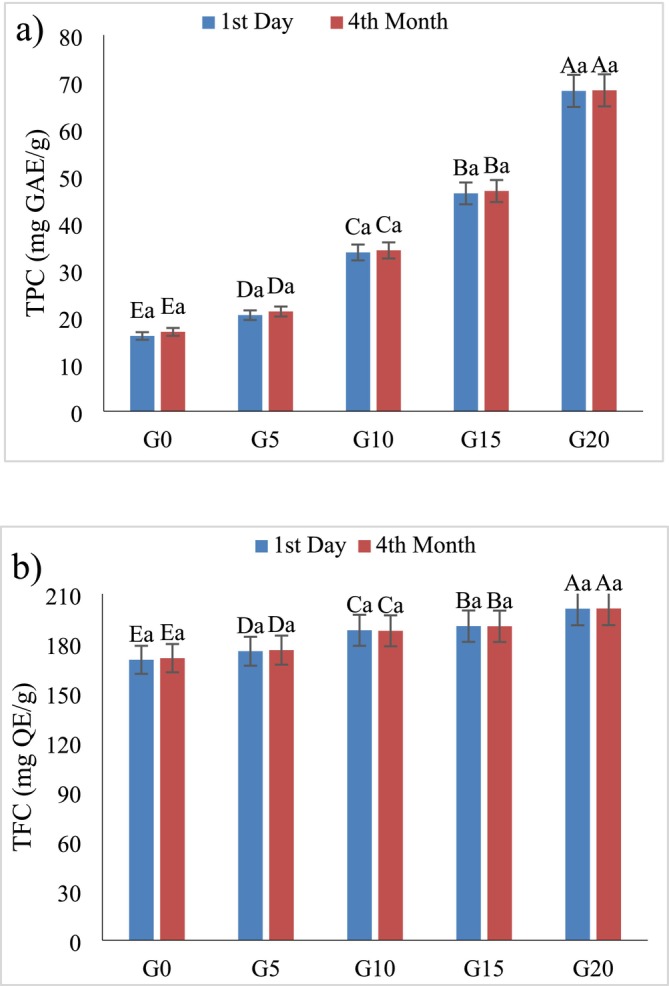
The effect of *G. lucidum* on TPC (a) and TFC (b) of functional chocolate during storage. Different uppercase letters indicate statistically significant differences (*p* < 0.05) between chocolates on the same day. Different lowercase letters indicate statistically significant differences (*p* < 0.05) between days of storage for chocolate. G0, G5, G10, G15, and G20 are chocolate formulations containing 0%, 5%, 10%, 15%, and 20% *G. lucidum* , respectively.

The TPC and TFC of functional chocolates varied across formulations and storage times (Figure 1a,b). Initially, an increase in GL concentration led to a rise in TPC, with the highest value observed in the G20 sample (68.23 mg GAE/g) and the lowest in G0 (16.0 mg GAE/g). During 4 months of storage, a slight growth in TPC was recorded in all samples. The observed enhancement in phenolic content can be attributed to polymerization and interactions with other chocolate matrix components, leading to potential production or structural modifications. However, the stability of TPC in most samples could be due to an optimal balance between GL bioactive compounds and the chocolate matrix, providing some protective effects. Kim et al. (2008) investigated the content of phenolic compounds in GL cultivated in Korea, which was found to be 162 mg/g dw (Kim et al. 2008).

Similarly, the TFC exhibited a parallel pattern to that of TPC. An increase in GL concentration led to a corresponding rise in the initial flavonoid levels, with the G20 sample recording the highest TFC (201.1 mg QE/g), and the control (G0) showing the lowest (170.0 mg QE/g). This enhancement is likely due to the abundance of semipolar flavonoid compounds naturally present in the fruiting body of *GL* (Mishra et al. 2018). During the 4‐month storage period, TFC values remained relatively stable across all samples, with only a slight increase observed. This suggests that flavonoid compounds in the chocolate matrix were more resistant to degradation compared to total phenolics, potentially due to their structural stability against oxidative stress and light exposure. Minor decreases in TFC observed in G10 and G15 samples may be attributed to mild oxidative reactions or photodegradation during storage. These results are consistent with previous findings. For instance, Sułkowska‐Ziaja et al. (2022) reported a TFC of 0.22 mg rutin equivalent/g in *G. lucidum* (Sułkowska‐Ziaja et al. 2022), whereas Mohammadifar et al. (2020) measured a TFC of 1.372 mg quercetin equivalent/g in Iranian samples (Mohammadifar et al. 2020). Similarly, Cilerdzic et al. (2016) documented TFC values ranging from 0.26 to 0.64 μg QE/mg (Cilerdzic et al. 2016). Tang et al. (2016) also confirmed the presence of both TPC and TFC in different *Ganoderma* species, including *G. lucidum* (red lingzhi) and *G. sinense* (purple lingzhi), supporting the diversity and richness of bioactive compounds in this medicinal mushroom (Tang et al. 2016).

### 3.3 Antioxidant Capacity and Anticancer Activity

**FIGURE 2:**
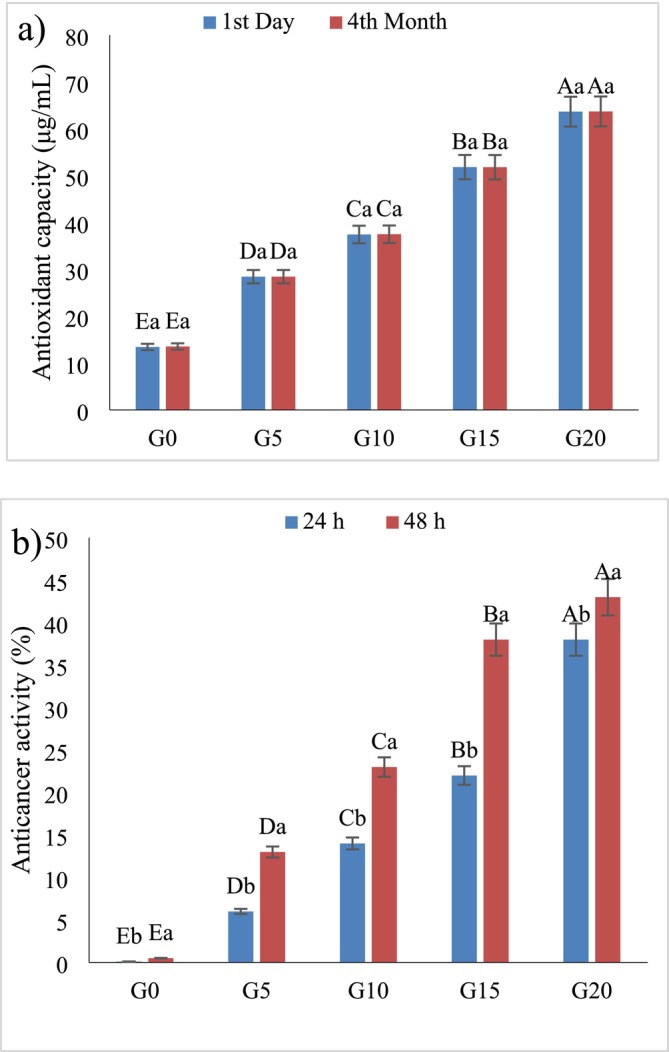
The effect of *G. lucidum* on antioxidant capacity (a) and antitumor activity (b) of functional chocolate during storage. Different uppercase letters indicate statistically significant differences (*p* < 0.05) between chocolates on the same day. Different lowercase letters indicate statistically significant differences (*p* < 0.05) between days of storage of chocolate. G0, G5, G10, G15, and G20 are chocolate formulations containing 0%, 5%, 10%, 15%, and 20% *G. lucidum* , respectively.

The antioxidant capacity of GL‐enriched chocolates showed a dose‐dependent increase (Figure 2a), with G20 exhibiting the highest initial activity (63.65 μg/mL) while the control (G0) had the lowest (13.43 μg/mL). Interestingly, antioxidant capacity slightly increased during 4 months of storage, possibly due to structural stabilization, bioconversion of precursors, and formation of active complexes between chocolate and GL bioactives. Additionally, interactions between different compounds in the system can lead to the formation of new complexes with higher antioxidant activity, contributing to the overall increase in antioxidant effects over time. Dehydration and cultivation processes could influence the TPC or TFC and antioxidant activity. For example, selenium‐rich substrate increases both TPC and the antioxidant power (Fraile‐Fabero et al. 2021). Similar findings have been reported by Kim et al. (2008), who noted a DPPH scavenging activity of approximately 70° for GL extracts (Kim et al. 2008). Veljovi et al., studied TPC and DPPH parameters. The TPC and DPPH values of six samples were between 8.6%–13.9% gallic acid equivalents and 2.08–3.07 (mM TE), respectively (Veljović et al. 2017). Chen et al. (2024) also confirmed that ultrafine grinding of Ganoderma with other medicinal food ingredients significantly boosts antioxidant effects by reducing oxidative stress and apoptosis.

The antitumor activity of functional chocolates, expressed as inhibition percentage, exhibited a remarkable increase over time (Figure 2b). On the 24 h, the G20 sample showed the highest inhibition rate (38%), which further increased to 43% after 48 h. Interestingly, all samples containing GL demonstrated enhanced antitumor activity with storage, whereas the control sample (G0) exhibited only a minimal improvement from 0.1% to 0.5%. This increasing trend can be attributed to several mechanisms, including the release and activation of bioactive compounds, where interactions between GL polysaccharides, triterpenes, and chocolate components during storage may enhance bioavailability and efficacy; the formation of more potent derivatives, as certain bioactive compounds in GL, particularly triterpenoids, might undergo transformations that amplify their cytotoxic effects against cancer cells; and increased synergistic effects, where prolonged interactions between polyphenols, flavonoids, and GL bioactives may result in enhanced cooperative effects, thereby strengthening antitumor activity. The stability of GL bioactives over time suggests that controlled storage conditions could potentially further enhance the therapeutic potential of the product. Moreover, the incorporation of GL into chocolate formulations significantly improved both antioxidant capacity and antitumor activity. Although antioxidant activity experienced a slight decline due to oxidation and structural changes, the increase in antitumor activity over time indicates that the bioactive compounds in GL might become more potent with storage. These findings underscore the potential of GL‐enriched chocolates as FFs with long‐term health benefits, reinforcing their role as an innovative platform for delivering health‐promoting bioactives with prolonged efficacy. By incrementing the percentage of GL, antitumor activity also increased. The presence of polysaccharides in GL can contribute to anticancer activity. GL can restrict the proliferation and the induction of apoptosis of tumor cells. Furthermore, it diminishes the integrin to prevent tumor cell adhesion (Lu et al. 2020). The antitumor and anticancer effects of GL mushroom have been proven in various studies (Barbieri et al. 2017; Martínez‐Montemayor et al. 2019; Sohretoglu and Huang 2018).

### 3.4 Chocolate's Oil Oxidation

Table 2 examines the effect of GL on oil oxidation in functional chocolates over 4 months of storage. Three key indicators—PV, TBARs, and AV—were analyzed to assess oil stability. The PV indicates the presence of primary oxidation products in fats. On the first day, all samples had a similar PV (0.14 mEq/kg), but after 4 months, PV increased in all samples. The highest increase was observed in the G0 sample (0.23 mEq/kg), whereas the G20 sample showed the lowest increase (0.17 mEq/kg). This reduction in PV increase in samples containing GL suggests that the mushroom plays a protective role in reducing the primary oxidation of chocolate lipids.

**TABLE 2:** The effect of *Ganoderma lucidum* on oil oxidation of functional chocolate during storage.

Parameters	Storage time	G0	G5	G10	G15	G20
PV (meq/kg)	1th day	0.14 ± 0.02^Ba^	0.14 ± 0.02^Bb^	0.14 ± 0.04^Bc^	0.14 ± 0.04^Bd^	0.14 ± 0.05^Be^
	4th month	0.23 ± 0.01^Aa^	0.19 ± 0.01^Ab^	0.18 ± 0.02^Ab^	0.18 ± 0.02^Ab^	0.17 ± 0.04^Ab^
TBARs (mg MDA/kg)	1th day	6.81 ± 0.2^Aa^	5.62 ± 0.05^Ab^	3.37 ± 0.02^Ac^	1.45 ± 0.0^Ad^	0.82 ± 0.04^Ae^
	4th month	6.78 ± 0.04^Aa^	5.98 ± 0.02^Ab^	4.10 ± 0.02^Ac^	1.49 ± 0.03^Ad^	0.87 ± 0.01^Ae^
Acidity (°D)	1th day	1.32 ± 0.05^Aa^	1.30 ± 0.02^Aa^	1.33 ± 0.02^Aa^	1.33 ± 0.02^Aa^	1.31 ± 0.03^Aa^
	4th month	1.40 ± 0.03^Aa^	1.41 ± 0.03^Aa^	1.40 ± 0.01^Aa^	1.44 ± 0.02^Aa^	1.42 ± 0.02^Aa^

*Note:* Different lowercase letters (Aa, Ab, Ac, Ad, Ae, Ba, Bb, Bc, Bd, Be) indicate statistically significant differences (*p* < 0.05) between chocolates at same day. Different uppercase letters indicate statistically significant differences (*p* < 0.05) between days of storage at chocolate. G0, G5, G10, G15, and G20 are chocolate formulations containing 0%, 5%, 10%, 15%, and 20% *G. lucidum* , respectively.

The TBARs index measures secondary oxidation products, particularly malondialdehyde (MDA). On the first day, the G0 sample (chocolate without GL) had the highest TBARs value (6.81 μg MDA/g), whereas the G20 sample had the lowest (0.82 μg MDA/g). This reduction demonstrates the antioxidant effect of GL in preventing lipid oxidation. After 4 months, minor changes in TBARs values were observed, but the samples containing GL still had significantly lower values than G0, indicating improved lipid stability in these formulations.

Acidity measures the hydrolysis of triglycerides and the release of free fatty acids, which can degrade fat quality. On the first day, acidity levels ranged from 1.3% to 1.33%, and after 4 months, only slight variations were detected. However, no statistically significant differences were observed between samples, indicating that GL had no substantial impact on this parameter.

Overall, the incorporation of GL effectively delayed both primary and secondary lipid oxidation in the chocolate, as evidenced by lower PV and TBARs values. This protective effect is likely due to the antioxidant action of polysaccharides and triterpenoids in GL. These results are in line with findings by Ghobadi et al. (2018), who reported enhanced oxidative stability in sausage emulsions with GL, and Nabati et al. (2023), who showed improved chemical and microbial preservation in fish using GL‐loaded packaging (Ghobadi et al. 2018; Nabati et al. 2023).

### 3.5 Color Indexes

Table 3 presents the impact of GL incorporation on the color parameters (L*, a*, b*) of functional chocolates over 4 months of storage. They were significantly influenced by GL addition. The L* value (lightness) decreased with increasing GL concentration, indicating a darker chocolate, which is attributed to the natural brown pigments in GL and possible Maillard reaction products formed during the drying of the mushroom powder. On the first day, the control sample (G0) had the highest L* value (31.8), whereas the G20 sample had the lowest (8.3), indicating a substantial darkening effect. After 4 months, slight variations were observed, but the trend remained consistent, suggesting that GL contributed to a darker chocolate color that remained stable over time, which was supported by a previous study conducted by Nguyen et al. (2019). They indicated that enhancing the amount of GL in wines resulted in reducing L* values (Nguyen et al. 2019).

**TABLE 3:** The effect of *Ganoderma lucidum* on color index and hardness of functional chocolate during storage.

Parameters	Storage time	G0	G5	G10	G15	G20
L*	1th day	31.8 ± 0.03^Aa^	26.4 ± 0.02^Ab^	20.2 ± 0.73^Ac^	19.30 ± 0.02^Ad^	8.3 ± 0.05^Ae^
	4th month	31.72 ± 0.02^Aa^	26.54 ± 0.4^Ab^	20.54 ± 0.12^Ac^	19.65 ± 0.57^Ad^	8.68 ± 0.5^Ae^
a*	1th day	74.20 ± 0.08^Ae^	79.5 ± 0.02^Ad^	81.54 ± 0.02^Ac^	94.7 ± 0.02^Ab^	104.2 ± 0.02^Aa^
	4th month	75.2 ± 0.04^Ae^	79.51 ± 0.05^Ad^	82.0 ± 0.01^Ac^	95.2 ± 0.12^Ab^	105.2 ± 0.06^Aa^
b*	1th day	12.4 ± 0.03^Aa^	9.4 ± 0.02^Ab^	5.2 ± 0.03^Ac^	4.1 ± 0.015^Ad^	1.98 ± 0.03^Ae^
	4th month	12.72 ± 0.02^Aa^	9.54 ± 0.01^Ab^	5.67 ± 0.02^Ac^	4.8 ± 0.57^Ad^	2.0 ± 0.03^Ae^
Hardness (N)	1th day	15.67 ± 0.02^Be^	24.54 ± 0.01^Bd^	30.52 ± 0.09^Bc^	40.81 ± 0.32^Bb^	53.20 ± 0.02^Ba^
	4th month	17.40 ± 0.07^Ae^	24.67 ± 0.03^Ad^	30.87 ± 0.01^Ac^	41.88 ± 0.26^Ab^	54.23 ± 0.05^Aa^

*Note:* Different lowercase letters (Aa, Ab, Ac, Ad, Ae, Ba, Bb, Bc, Bd, Be) indicate statistically significant differences (*p* < 0.05) between chocolates at same day. Different uppercase letters indicate statistically significant differences (*p* < 0.05) between days of storage at same chocolate. G0, G5, G10, G15, and G20 are chocolate formulations containing 0%, 5%, 10%, 15%, and 20% *G. lucidum* , respectively.

The a* value, associated with redness, increased with GL concentration. The G20 sample exhibited the highest a* value (104.2) on the first day, whereas the G0 sample had the lowest (74.2). After 4 months, all samples showed slight increases in a* values, with G20 reaching 105.2, indicating that the red pigmentation of GL remained stable or slightly intensified during storage.

The b* value, representing yellowness, decreased as GL concentration increased. The G0 sample had the highest b* value (12.4) on the first day, whereas G20 had the lowest (1.98). Over 4 months, minor fluctuations were observed, but the general trend persisted, demonstrating that GL contributed more to red and dark hues rather than yellow tones.

The addition of GL significantly altered the color properties of functional chocolate, making it darker and redder with increasing concentration. These changes remained stable throughout storage, highlighting the color‐enhancing effects of GL due to its bioactive compounds. This suggests that GL‐enriched chocolates may exhibit visually distinct characteristics while maintaining their appearance over time.

### 3.6 Hardness Analysis of Chocolate

One of the most important properties of chocolate is hardness, which is described as the point where the construction is deformed by an applied force (Toker et al. 2018). The incorporation of GL significantly influenced the hardness of functional chocolates over 4 months of storage (Table 3). The hardness increased with higher concentrations of GL, as observed on the first day when the control sample (G0) had the lowest hardness (15.67 N) while G20 exhibited the highest (53.20 N). Over time, all samples became firmer, with G0 reaching 17.40 N and G20 increasing to 54.23 N. This trend suggests that GL contributes to a denser chocolate matrix through various mechanisms, including polysaccharide‐lipid interactions, which may influence lipid crystallization; moisture redistribution, leading to increased firmness; and protein‐polyphenol interactions, which enhance structural integrity (Aravind et al. 2012; Brennan et al. 2013). Overall, the presence of GL significantly improved the textural stability of chocolate, making it firmer over time and potentially extending its shelf life. This is due to the ability of laccase in GL to cross‐link chocolate proteins. Therefore, cross‐linkage of protein catalyzed by laccase is one of the main reasons for the enhancement of chocolate hardness (Guowei et al. 2019). Previous studies also reported an increasing trend in the hardness of foodstuff by the addition of mushroom powder (Lu et al. 2016).

### 3.7 Sensory Results

**FIGURE 3:**
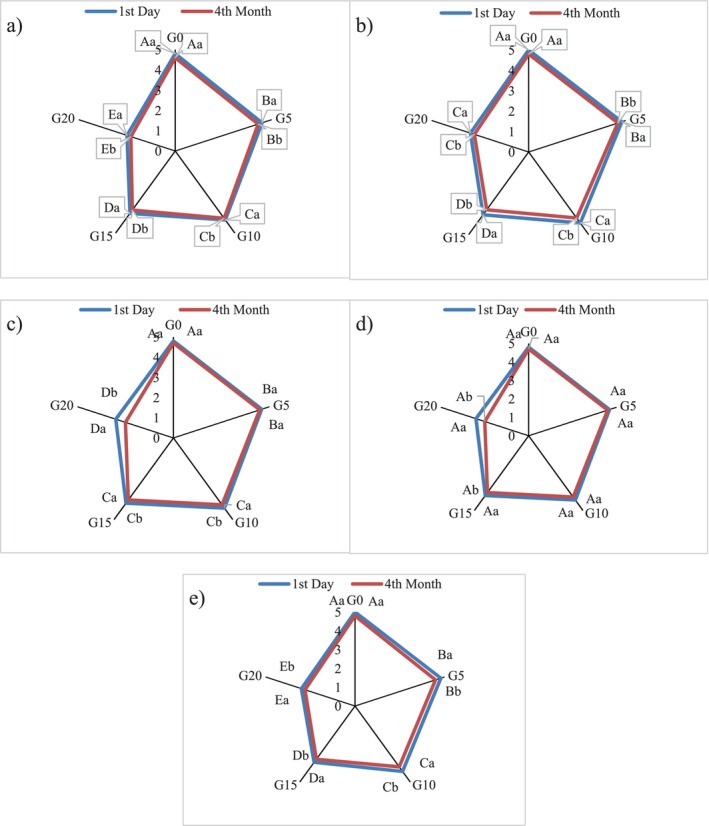
The effect of *G. lucidum* on sensory properties (a) texture, (b) color, (c) melting in month, amount of sweets (d) and overall acceptance (e) of functional chocolate during storage. Different uppercase letters indicate statistically significant differences (*p* < 0.05) between chocolates on the same day. Different lowercase letters indicate statistically significant differences (*p* < 0.05) between days of storage of chocolate. G0, G5, G10, G15, and G20 are chocolate formulations containing 0%, 5%, 10%, 15%, and 20% *G. lucidum* , respectively.

The sensory evaluation of functional chocolates enriched with GL was assessed based on texture, color, melting in the mouth, sweetness, and overall acceptance over 4 months of storage (Figure 3a,b). The results indicate a general decline in sensory attributes with increasing GL concentration and storage time.

The hardness of the chocolates increased with higher GL concentrations, which negatively impacted the perceived texture (Figure 3a). On the first day, G0 had the highest texture score (4.8), whereas G20 had the lowest (2.5). After 4 months, all samples showed a slight decline, with G0 decreasing to 4.6 and G20 to 2.3. This suggests that the firmness associated with GL led to a less favorable texture perception.

Color acceptability also decreased with increasing GL levels, as the chocolates became darker (Figure 3b). G0 had the highest score (5.0), whereas G20 had the lowest (3.0) on the first day. By the fourth month, G0 remained the most preferred (4.8), whereas G20 further declined (2.8), indicating that darker chocolates were less appealing to panelists.

Chocolates with higher GL content had lower melting in the mouth and sweetness scores (Figure 3c) likely due to the interaction between polysaccharides and chocolate lipids. G0 had the best melting sensation (4.8) and the highest sweetness perception (4.8) on the first day, whereas G20 had the lowest (3.0 for both). Over time, all samples experienced a slight decrease, with G20 dropping to 2.5, reinforcing that higher GL concentrations negatively impacted mouthfeel and sweetness perception. This reduction of melting in the mouth may be due to the higher fat content of chocolates, as well as lower viscosity, making them easier to flow (Tan and Kerr 2018).

The overall acceptance score followed the same trend (Figure 3d), with G0 receiving the highest rating (5.0) and G20 the lowest (3.0) on the first day. By the fourth month, G0 still had the best acceptance (4.8), whereas G20 decreased further (2.8). This indicates that while GL offers functional benefits, its impact on sensory attributes, especially texture and sweetness, reduces consumer preference.

The incorporation of GL led to a darker, firmer, and less sweet chocolate, which negatively affected sensory acceptance. Although these chocolates provide potential health benefits, optimizing the formulation to balance functionality and sensory attributes is crucial for consumer satisfaction. Previous studies conducted by Ghobadi et al. (2018) and Wannasupchue et al. (2011) also reported that GL addition reduces consumer sensory satisfaction for different types of sausages (Ghobadi et al. 2018; Wannasupchue et al. 2011).

## 4 Conclusion

The incorporation of *GL* into chocolate significantly improved its functional properties, particularly antioxidant and antitumor activities, with relatively good bioactive stability during storage. However, increasing GL concentration adversely affected sensory characteristics such as texture, color, and sweetness, leading to decreased consumer acceptance. These results suggest that GL‐enriched chocolates could be promising FFs, but formulation optimization is essential to balance health benefits and sensory quality. Future research should explore strategies to improve sensory attributes, such as combining GL with flavor enhancers or texture‐modifying agents, whereas preserving its bioactivity.

## Author Contributions

**Yasamin Zahra Bazyar:** conceptualization (equal), data curation (equal), formal analysis (equal), investigation (equal), methodology (equal), writing – original draft (equal). **Mohammad Rabbani:** supervision (equal), validation (equal), visualization (equal), writing – original draft (equal), writing – review and editing (equal). **Mohammad Hossein Azizi:** methodology (equal), project administration (equal), visualization (equal), writing – original draft (equal), writing – review and editing (equal).

## Conflicts of Interest

The authors declare no conflicts of interest.

## Data Availability

Data will be made available on request.
